# An observational and Mendelian randomisation study on vitamin D and COVID-19 risk in UK Biobank

**DOI:** 10.1038/s41598-021-97679-5

**Published:** 2021-09-14

**Authors:** Xue Li, Jos van Geffen, Michiel van Weele, Xiaomeng Zhang, Yazhou He, Xiangrui Meng, Maria Timofeeva, Harry Campbell, Malcolm Dunlop, Lina Zgaga, Evropi Theodoratou

**Affiliations:** 1 School of Public Health and the Second Affiliated Hospital, Zhejiang University School of Medicine, Hangzhou, China; 2 Centre for Global Health, Usher Institute, University of Edinburgh, Edinburgh, Scotland, UK; 3 Royal Netherlands Meteorological Institute (KNMI), De Bilt, The Netherlands; 4 Vanke School of Public Health, Tsinghua University, Beijing, 10084 China; 5 Colon Cancer Genetics Group, Cancer Research UK Edinburgh Centre and Medical Research Council Human Genetics Unit, Medical Research Council Institute of Genetics and Molecular Medicine, University of Edinburgh, Edinburgh, UK; 6 DIAS, Danish Institute for Advanced Study, Department of Public Health, University of Southern Denmark, Odense, Denmark; 7 Department of Public Health and Primary Care, Institute of Population Health, Trinity College Dublin, Dublin, Republic of Ireland; 8 Cancer Research UK Edinburgh Centre, MRC Institute of Genetics and Molecular Medicine, University of Edinburgh, Edinburgh, Scotland

**Keywords:** Risk factors, Nutrition

## Abstract

A growing body of evidence suggests that vitamin D deficiency has been associated with an increased susceptibility to viral and bacterial respiratory infections. In this study, we aimed to examine the association between vitamin D and COVID-19 risk and outcomes. We used logistic regression to identify associations between vitamin D variables and COVID-19 (risk of infection, hospitalisation and death) in 417,342 participants from UK Biobank. We subsequently performed a Mendelian Randomisation (MR) study to look for evidence of a causal effect. In total, 1746 COVID-19 cases (399 deaths) were registered between March and June 2020. We found no significant associations between COVID-19 infection risk and measured 25-OHD levels after adjusted for covariates, but this finding is limited by the fact that the vitamin D levels were measured on average 11 years before the pandemic. Ambient UVB was strongly and inversely associated with COVID-19 hospitalization and death overall and consistently after stratification by BMI and ethnicity. We also observed an interaction that suggested greater protective effect of genetically-predicted vitamin D levels when ambient UVB radiation is stronger. The main MR analysis did not show that genetically-predicted vitamin D levels are causally associated with COVID-19 risk (OR = 0.77, 95% CI 0.55–1.11, P = 0.160), but MR sensitivity analyses indicated a potential causal effect (weighted mode MR: OR = 0.72, 95% CI 0.55–0.95, P = 0.021; weighted median MR: OR = 0.61, 95% CI 0.42–0.92, P = 0.016). Analysis of MR-PRESSO did not find outliers for any instrumental variables and suggested a potential causal effect (OR = 0.80, 95% CI 0.66–0.98, p-val = 0.030). In conclusion, the effect of vitamin D levels on the risk or severity of COVID-19 remains controversial, further studies are needed to validate vitamin D supplementation as a means of protecting against worsened COVID-19.

## Introduction

A growing body of evidence shows that vitamin D deficiency might be associated with an increased susceptibility to viral and bacterial respiratory infections^1–3^. Similar findings have been recently reported for COVID-19: by analysing publicly available patient data, researchers have found a strong correlation between vitamin D deficiency and COVID-19 risk^4^. Furthermore, evidence suggests that COVID-19 disproportionately affects black and minority ethnic individuals, with one potential explanation being the higher prevalence of vitamin D deficiency, in addition to other risk factors^5^. It is thus hypothesised that having adequate vitamin D levels may help reduce the risk of contracting the SARS-CoV-2 virus or reduce the risk of severe or lethal COVID-19 disease.

To explore the causal role of vitamin D in COVID-19 risk, there have been at least three Mendelian Randomisation studies using the genetic variants associated with serum 25OHD as instrumental variables^6–8^. It is shown that genetic predisposition for lower levels of vitamin D is not causally associated with infection from SARS-CoV-2 or severe COVID-19 disease^6–9^. It is important to note that genetic heritability of vitamin D status is high in winter, but in the summer the vitamin D status might be predominantly determined by environmental factors regulating exposure (including season, geographical latitude) to ultraviolet B (UVB) radiation^10^. Therefore, an integrative measure of both genetically and ambient UVB radiation determined vitamin D levels during the pandemic would provide comprehensive insight in the causal inference in relation to vitamin D and COVID-19 risk.

The main aim of the current study is to perform Mendelian Randomisation (MR) analyses investigating the effect of genetically-predicted vitamin D levels on COVID-19 risk while taking into account ambient UVB radiation at the time of the infection, and compare these findings with results obtained from the observational analysis. We firstly conducted an observational study to examine the associations between measured vitamin D levels and COVID-19 risk. We then performed a MR analysis by using genetically-predicted vitamin D levels and also applied a novel approach that enabled us to estimate the UVB exposure preceding disease onset to COVID-19 to account for seasonal differences.

## Methods

### Data sources

Basic demographic information and genotype data on 495,780 participants from UK Biobank^11^, a large prospective study, were linked to COVID-19 test results (for the period 16/03/2020 to 29/06/2020 provided by Public Health England), including the specimen date, origin (whether the person was an inpatient or not) and result (positive or negative), and death cases caused by clinically and epidemiologically diagnosed COVID-19 from death registry. Confirmed COVID-19 cases were defined as UK Biobank participants who had at least one positive test result or died of COVID-19. Participants who have not been tested for SARS-CoV-2 were taken as controls. We additionally excluded the following participants from the cohort: (1) those who tested negative, since test results could have been false negative; (2) participants, who were from Scotland and Wales, since all COVID-19 test results were provided by NHS England only; (3) participants who died before 01/01/2020, since they had no chance to be infected by SARS-COV-2. Total plasma 25-hydroxy-vitamin D (25-OHD) was measured at the baseline assessment visits between 2006 and 2010 (median of 11 years before COVID-19 pandemic), using immunoassay (Diasorin). To remove the effect of sampling season on 25-OHD levels, we generated May-standardised 25-OHD levels for all participants (approximating 25-OHD concentration if blood was drawn in May), by applying coefficients generated in a model restricted to controls and adjusted for age and sex^12^. Vitamin D status was further categorised as deficient (25-OHD < 25 nmol/L), insufficient (25–50 nmol/L), or sufficient (> 50 nmol/L). A total of 138 genetic variants have recently been reported to be associated with vitamin D from the largest Genome Wide Association Study (GWAS; n = 443,734)^13^. We excluded ambiguous AT and CG variants (n = 4, rs184958517, rs200641845, rs529640451, rs536006581) to avoid bias due to strand differences between studies, and finally 134 SNPs were selected as genetic instruments for the MR analysis. The effects of vitamin D SNPs on COVID-19 outcomes were examined in the UK Biobank participants of White ancestry only to minimize the influence of population structure. A weighted genetic risk score (wGRS) was calculated as a proxy of genetically-predicted 25-OHD levels for a life-long exposure in the UK Biobank White population by using effect estimates reported by Manousaki et al.^13^. Dermal synthesis following exposure to UVB radiation is a major source of vitamin D for humans. We used ambient UVB radiation to approximate vitamin D status attributable to dermal synthesis (vitD-UVB) at the time of COVID-19 diagnosis. To do this, we calculated the cumulative and weighted vitD-UVB dose form the TEMIS database, version 2.0 (http://www.temis.nl/uvradiation/UVdose.html). Briefly, we extracted daily UVB dose at wavelengths that induces vitamin D synthesis at each participant’s residential location over 135 days preceding the date of diagnosis for cases. Dates were randomly allocated to controls, from the distribution that was identical to that observed in cases. We weighted the daily UVB contributions before summing them up because more recent UVB exposure contributes more than exposures from a more distant past, since vitamin D is being synthesized and used up. More details on the calculation are presented elsewhere^14–16^ and in “Supplementary Method”.

Considering vitamin D receptor (VDR) may modify the biological effects of vitamin D, five variants (rs7975232, rs1544410, rs2228570, rs731236 and rs11568820) that are associated with VDR function were tested for any effect modification by adding multiplicative interaction terms in logistic regression model to examine whether the carrier of genetic polymorphisms of VDR would modify the effect of vitamin D on COVID-19 risk.

### Statistical analysis

In the descriptive analysis, mean and Standard Deviation (SD) is given for continuous variables, and number (N) and proportion for categorical variables, unless indicated otherwise. Logistic regression modelling was used to estimate the effect of vitamin D variables on COVID-19 risk (the risk of infection, hospitalisation and death) after adjustment for a range of covariates, including age, sex, deprivation index, body mass index (BMI), month of blood draw, ethnicity, physical activity, smoking and alcohol status, sunshine exposure variables, vitamin D supplement intake, and comorbidities of cardiovascular diseases (CVDs), diabetes, asthma, and malignancy. Specifically, we investigated the associations between COVID-19 and: (1) vitamin D levels (circulating 25-OHD concentration, May-standardised 25-OHD concentration, and categorical vitamin D status); (2) vitD-UVB, an integrated measure of ambient UVB radiation during the pandemic; (3) genetically-predicted 25-OHD concentration using wGRS (vitD-wGRS_134_), in fully adjusted models as described above. When analysing the association between vitD-wGRS_134_ and COVID-19 risk, we additionally adjusted for the first 20 genetic principal components and genotyping panel to account for any potential confounding caused by population structure. Bonferroni correction was applied to account for multiple testing. As we tested the associations between five vitamin D variables (vitD levels, vitD-May-adjusted, vitD-categorical, vitD-UVB and vitD-wGRS_134)_ and three COVID-19 outcomes (risk of infection, hospitalisation, and death), we adjusted the significant threshold as p < 0.003 (0.05/15). We also tested their interactions with VDR SNPs. For MR analyses, vitamin D SNPs were aligned by the vitamin D increasing alleles, and the genetic associations between vitamin D SNPs and COVID-19 infection risk were estimated with adjustment for age, sex, the first 20 genetic PCs and genotype panel. Inverse-variance weighted (IVW) MR approach was used as the main analysis, and the simple mode, Egger, weighted median, weighted mode and MR-Pleiotropy RESidual Sum and Outlier (MR-PRESSO) as sensitivity analyses to explore the robustness of the findings in the presence of potential pleiotropy of the genetic variants^17^. The statistical power of MR analysis was calculated by using the non-centrality parameter-based approach^18^, and the overall proportion of variance (R^2^) of vitamin D levels explained by the genetic instruments was estimated by using the measured vitamin D levels in the study population. Details of these MR approaches, including their different assumptions, are provided in “Supplementary Methods” and elsewhere^19,20^. All analyses were conducted using R version 3.6.1.

### Ethics approval and consent to participate

UK Biobank has approval from the North West Multi-Centre Research Ethics Committee (11/NW/0382) and obtained written informed consent from all participants prior to the study. No consent to participate was required.

### Ethical statement

The co-authors confirm that all methods in this study were carried out in accordance with relevant guidelines and regulations.

## Results

There was a total of 14,439 COVID-19 tests conducted in UK Biobank participants. Of these, 1596 individuals had at least one positive COVID-19 test and 1020 of them were hospitalised. Additional 399 COVID-19 death cases were identified from the death registry. Table 1 presents the basic demographic characteristics of the cohort. In multivariate regression analysis, vitD-UVB at recruitment was strongly associated with 25OHD concentrations at recruitment (beta = 0.11, p-val < 2 × 10^–16^, R^2^ = 0.19). The variance of 25OHD concentration at recruitment explained by vitD-UVB at recruitment alone was 12.4%, by vitD-GRS_134_ alone was 4.2%, and by vitD-GRS_134_ and vitD-UVB together with covariates in a multivariate model was 23.1%. Given the number of COVID-19 patients and the percentage of variance (4.2%) explained by vitamin D-related genetic variants, the main MR analysis was adequately powered (> 80%) to detect moderate to large causal effect with an odds ratio (OR) less than 0.68 (or greater than 1.32) per SD change in standardized natural-log transformed 25OHD levels.

**Table 1:** Baseline characteristics of the COVID-19 cases and controls in UK Biobank.

	COVID-19 cases			Total cases (n = 1746)	Controls (n = 415,596)
	Outpatient (n = 576)	Inpatient (n = 1020)	Death (n = 399)		
Gender, N (%)	Male: 264 (45.8%)	Male: 570 (55.9%)	Male: 255 (63.9%)	Male: 924 (52.9%)	Male: 185,494 (44.6%)
	Female: 312 (54.2%)	Female: 450 (44.1%)	Female: 144 (36.1%)	Female: 822 (47.1%)	Female: 230,102 (55.4%)
Age, Mean (SD)	65.97 (9.42)	69.40 (8.86)	74.66 (5.98)	68.76 (9.18)	68.14 (8.08)
BMI (kg/m^2^)	27.66 (5.01)	27.47 (4.81)	27.59 (4.68)	27.53 (4.88)	27.41 (4.80)
vitD (nmol/l), median (IQR)	46.11 (30.20–59.30)	46.82 (29.40–61.35)	44.30 (29.70–60.42)	46.57 (29.70–60.83)	47.00 (32.60–62.60)
vitD-UVB (kJ/m^2^), median (IQR)^a^	90.80 (67.43–98.95)	48.96 (33.81–81.08)	43.09 (31.89–74.10)	65.95 (38.10–99.08)	66.25 (37.63–100.52)
Vitamin D supplement, N (%)	21 (3.6%)	43 (4.2%)	12 (3.0%)	64 (4.0%)	17,764 (4.3%)
**Smoking status, N (%)**					
Never	318 (55.2%)	462 (45.3%)	148 (37.1%)	780 (48.9%)	231,192 (55.6%)
Previous	190 (33.0%)	429 (42.0%)	186 (46.6%)	619 (38.8%)	140,876 (33.9%)
Current	63 (10.9%)	116 (11.4%)	60 (15.0%)	179 (11.2%)	41,182 (9.9%)
Unknown	5 (0.9%)	13 (1.3%)	5 (1.3%)	18 (1.1%)	2346 (0.6%)
**Alcohol drinker status, N (%)**					
Never	45 (7.8%)	79 (7.7%)	30 (7.5%)	124 (7.8%)	18,341 (4.4%)
Previous	22 (3.8%)	61 (6.0%)	31 (7.8%)	83 (5.2%)	13,975 (3.4%)
Current	508 (88.2%)	873 (85.6%)	334 (83.7%)	1381 (86.5%)	382,064 (91.9%)
Unknown	1 (0.2%)	7 (0.7%)	4 (1.0%)	8 (0.5%)	1216 (0.3%)
**Ethnicity, N (%)**					
White	497 (86.3%)	883 (86.6%)	357 (89.5%)	1524 (87.2%)	390,739 (94.0%)
Asian	21 (3.6%)	56 (5.5%)	11 (2.8%)	78 (4.5%)	9538 (2.3%)
Black	17 (3.0%)	28 (2.7%)	12 (3.0%)	45 (2.6%)	3854 (0.9%)
Other/unknown	41 (7.1%)	53 (5.2%)	19 (4.7%)	99 (5.7%)	11,465 (2.8%)
**Month (of COVID-19 diagnosis)**					
March	47 (8.2%)	257 (25.2%)	114 (28.6%)	328 (18.8%)	78,547 (18.9%)^a^
April	242 (42.0%)	563 (55.2%)	228 (57.1%)	882 (50.5%)	209,460 (50.4%)^a^
May	218 (37.8%)	161 (15.8%)	49 (12.3%)	420 (24.1%)	99,743 (24.0%)^a^
June	69 (12.0%)	39 (3.8%)	8 (2.0%)	116 (6.6%)	27,845 (6.7%)^a^

^a^Dates were randomly allocated to controls for the calculation of vitD-UVB based on the distribution that was identical to that observed in cases.

Results from multivariable logistic regression models (Table 2) showed no significant associations between COVID-19 infection and vitamin D levels both crude (OR = 1.00, 95% CI 0.99–1.01, p-val = 0.593) and May-standardised 25OHD concentration (OR = 1.00, 95% CI 0.99–1.01, p-val = 0.592). Only a nominally significant association (p-val < 0.05) was found among white individuals who were vitamin D sufficient compared to vitamin D deficient (OR = 0.82, 95% CI 0.68–0.99, p-val = 0.036) (Table S1), however, it was not statistically significant after Bonferroni correction (p-val > 0.003). Neither vitD-UVB nor genetically-predicted (vitD-GRS_134_) vitamin D were associated with COVID-19 infection risk. In order to investigate whether vitamin D levels would influence COVID-19 severity, we performed a sensitivity analysis for hospitalised cases and COVID-19 deaths. We consistently found that vitD-UVB dose was strongly and inversely associated with the hospitalization (OR = 0.98, 95% CI 0.97–0.99, p-val < 2 × 10^–16^) and death (OR = 0.97, 95% CI 0.96–0.98, p-val < 2 × 10^–16^) from COVID-19 in multivariable models, while null findings were reported for other vitamin D variables (Table 2). Similar results were observed when we stratified the cohort by BMI (< 25 or ≥ 25) (Table S2). When stratifying the study population by ethnicity, we found that lower vitD-UVB was associated with increased risk of hospitalization and death of COVID-19 among White population in multivariable models (Table S1). Additionally, in White population, the risk of hospitalisation was around 38% lower in vitamin D sufficient individuals than those who were deficient in multivariable (OR = 0.62, 95% CI 0.41–0.94, p-val = 0.024) model, while these associations were not statistically significant after Bonferroni correction (p-val > 0.003).

**Table 2:** Association between Vitamin D and COVID-19 risk in multivariable regression models.

	COVID positive (N = 1746) *vs* controls (N = 415,596)		COVID hospitalization (N = 1020) *vs* non- hospitalization (N = 576) cases		COVID death (N = 399) *vs* non-death (N = 1347) cases	
	OR (95% CI)	*p-val*	OR (95% CI)	*p-val*	OR (95% CI)	*p-val*
vitD (nmol/l)^a^	1.00 (0.99–1.01)	0.593	1.00 (0.99–1.01)	0.506	1.00 (0.99–1.01)	0.356
vitD_May_adjusted (nmol/l)^a^	1.00 (0.99–1.01)	0.592	1.00 (0.99–1.01)	0.674	1.00 (0.99–1.01)	0.324
**vitD_categorical**^**a**^						
0–25 nmol/L	Ref	–	Ref	–	Ref	–
25–50 nmol/L	1.03 (0.87–1.20)	0.743	0.94 (0.64–1.39)	0.759	0.93 (0.57–1.52)	0.774
50 nmol/L	0.97 (0.81–1.16)	0.762	0.90 (0.63–1.28)	0.551	0.92 (0.54–1.56)	0.757
vitD-wGRS_134_^b^	0.91 (0.80–1.03)	0.134	1.05 (0.81–1.36)	0.721	1.25 (0.90–1.73)	0.175
vitD-UVB	1.00 (0.99–1.01)	0.557	0.98 (0.97–0.99)	< 2 × 10^–16^	0.97 (0.96–0.98)	< 2 × 10^–16^
**vitD-wGRS**_**134**_** + vitD-UVB**^**c**^						
vitD-wGRS_134_	0.92 (0.81–1.04)	0.191	0.88 (0.66–1.17)	0.391	1.12 (0.80–1.57)	0.508
vitD-UVB	1.00 (0.99–1.01)	0.511	0.98 (0.97–0.99)	< 2 × 10^–16^	0.97 (0.96–0.98)	4.35 × 10^–16^

^a^Adjusted for age, gender, body mass index (BMI), month of blood draw (adjusted for vitD and vitD-categorical only), ethnicity, physical activity, smoking and alcohol status, sunshine exposure variables (i.e., time spend outdoors in summer, time spent outdoors in winter and the use of sun/uv protection), vitamin D supplement intake, deprivation index, and comorbidities of CVDs, diabetes, asthma, and malignancy.

^b^Multivariable model was additionally adjusted for the first 20 genetic principal components and genotype panel.

^c^Multivariable regression was fitted by including both vitD-wGRS_134_ and vitD-UVB in the same model to examine the effects of genetically predicted vitamin D levels and ambient UVB jointly.

VitD-UVB was also marginally associated with hospitalisations in Asians (p-val = 0.044) and significantly associated with COVID-19 risk in Black population (p-val = 0.001) (Tables S3, S4). We observed no evidence of interaction between vitD-GRS_134_ and VDR SNPs, or between vitD-UVB and VDR SNPs (Table S5), while evidence of significant interaction was observed between vitD-GRS_134_ and vitD-UVB (beta = − 0.005, p-val = 0.001) for COVID-19 infection risk (Table S6). When stratified by vitD-UVB tertiles, the beta-coefficient for vitD-wGRS_134_ changed from 0.186 (p-val = 0.141) in Tertile 1, to − 0.173 (p-val = 0.125) in Tertile 2 and was statistically significant in Tertile 3 (beta = − 0.321, p-val = 0.003, Table S7), suggesting greater protective effect of genetic factors when ambient UVB is greater.

**Figure 1:**
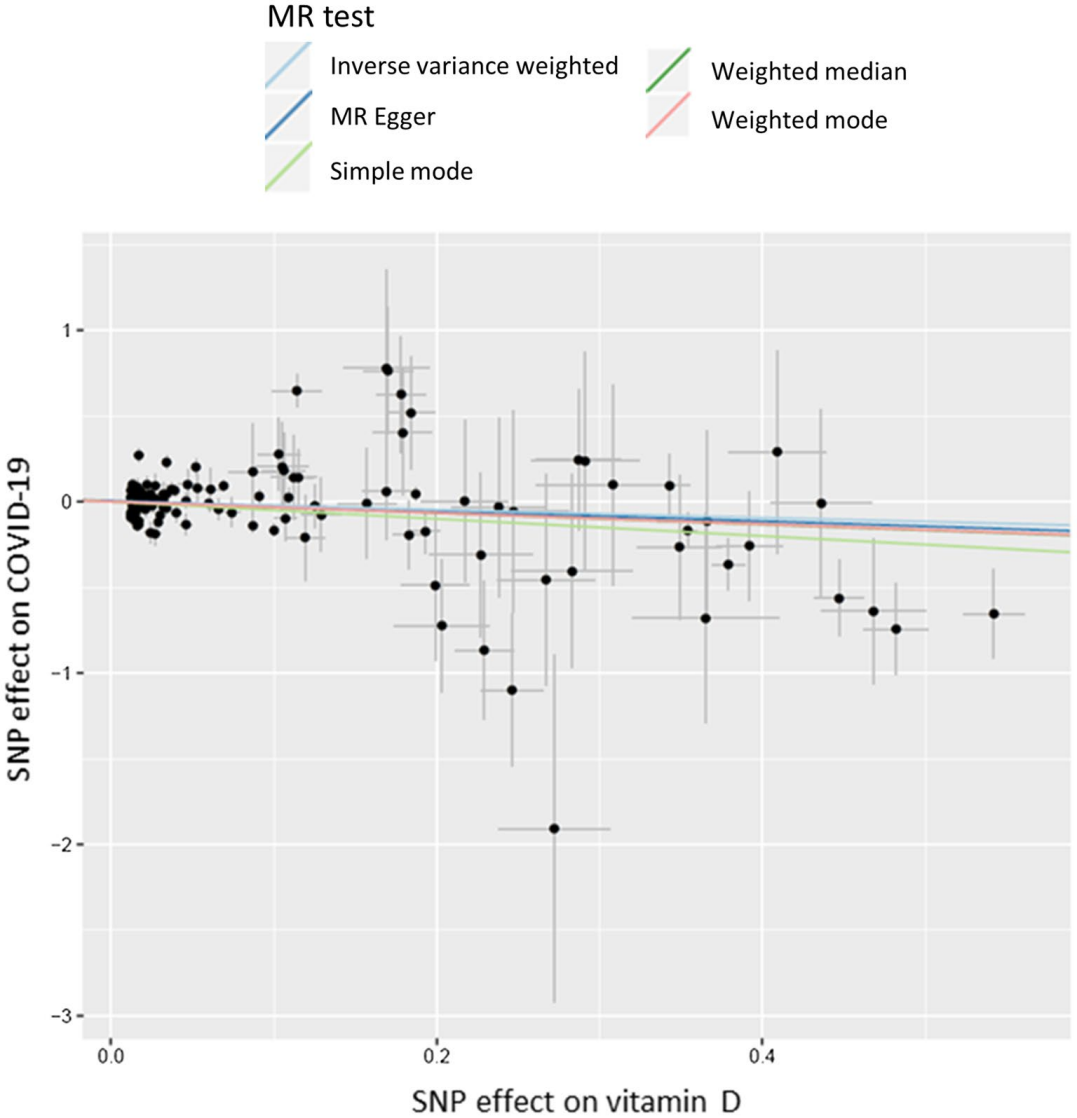
A scatter plot of Mendelian Randomisation analyses of 134 vitamin D SNPs on COVID-19 risk.

Table S8 shows the results of the MR analyses using 134 genetic instruments, which are also graphically presented in Fig. 1. The main IVW MR did not show causal association between genetically-predicted vitamin D levels and COVID-19 infection (OR = 0.77, 95% CI 0.55–1.11, p-val = 0.160), however the sensitivity analyses of weighted mode MR and weighted median MR suggested a potential causal effect (weighted mode MR: OR = 0.72, 95% CI 0.55–0.95, p-val = 0.021; weighted median MR: OR = 0.61, 95% CI 0.42–0.92, p-val = 0.016). Analysis of MR-PRESSO did not find outliers for any instrumental variables and indicated a potential causal effect (OR = 0.80, 95% CI 0.66–0.98, p-val = 0.030). The pleiotropy test from MR Egger analysis also indicated low likelihood of pleiotropy with non-significant intercept (P__Intercept_ = 0.161).

## Discussion

In this study, we assessed whether there is an association between vitamin D and COVID-19 risk and severity by examining a comprehensive set of key vitamin D variables jointly for the first time, and applying a number of analyses to probe consistency of our findings. We consistently found a strong inverse association between an integrated measure of ambient UVB preceding disease onset (vitD-UVB) and disease severity.

We, unsurprisingly, found no strong association between vitamin D levels (plasma 25-OHD concentration measured at recruitment, 11 years ago) and COVID-19 risk or severity after adjustment for confounders, results that are in accordance to the recent study by Hastie et al.^21^. In this cohort, vitD-UVB explained the largest portion of the variance in 25-OHD at recruitment: vitD-UVB alone explained 12.4%, while vitD-GRS_134_ alone explained 4.2%. Previous studies have shown that heritability of 25-OHD is high in winter and low in summer, which suggests a varied role of genetic factors, dependant on the UVB intensity^10^. It is therefore not surprising that we found evidence of an interaction between vitD-UVB and vitamin D genetic risk score, and these findings highlight the added value of examining genetically-predicted levels and ambient UVB jointly. MR sensitivity analyses using the weighted median and mode methods indicated a potential causal effect, although the main MR analysis showed that genetically-predicted vitamin D levels were not causally associated with COVID-19 risk.

UK Biobank is a large prospective study, with rich information on a range of demographic, lifestyle and health-related risk factors. Vitamin D plasma measurements were conducted in a single central processing laboratory using the Diasorin immunoassay, albeit a blood sample was taken over a decade ago and is unlikely to be representative of participants’ vitamin D status at the time of the pandemic. We have partially addressed this by using genetic instruments (that are determined by DNA sequence and hence not variable) to derive genetically-predicted vitD levels. It is important to note that heritability of vitamin D status is high in winter (70–90%), but levels might be entirely determined by environmental factors in the summer^10^. Therefore, we also included an integrative measure of ambient UVB radiation during the pandemic. Vitamin D status is highly correlated with numerous factors, many of which are also linked with poorer health. By using genetically-predicted vitamin D level MR approach offers a unique opportunity to bypass confounding originating from these associations. However, the vitamin D status is varying seasonally, due to the overpowering effect of solar radiation and dermal production it induces. To account for these seasonal differences, we have used a novel approach that enabled us to estimate the UVB exposure preceding disease onset. One of the key strengths of this study is that we included this covariate in the analysis, with and without modelling the interaction, which enabled us to account for the time-varying nature of the relationship that is commonly a major issue for vitamin D MR studies.

The discriminatory power of the UVB variable is somewhat limited in this study, because UVB radiation is low at this time of the year, particularly at the high northern latitude of UK—larger effects might be observed if variation in UVB is greater. We only used ambient UVB, and did not capture individual behavioural differences that would determine the actual level of vitamin D synthesis in the skin, such as duration and time of day spent outside, clothing, etc. It is important to note that time of year is the strongest predictor of vitD-UVB. To avoid bias control dates were assigned to follow the same distribution as case dates, which might have led to artificially diminished differences in vitD-UVB between cases and controls, however analysis relating to hospitalisation and death are not affected by this. We also conducted an analysis of the genetically-predicted vitamin D and a number of state-of-the-art MR analyses. However, the main limitation is the lack of power. Given the small number of COVID-19 patients and the relatively small percentage of variance (4.2%) explained by vitamin D-related genetic variants, this MR study was not adequately powered to detect small causal effect and negative results should be interpreted with caution. Additionally, MR studies only consider linear effects between 25-OHD levels and COVID-19 risk, which do not capture what happens at the extremes of vitamin D deficiency. Therefore, it cannot rule out the possibility that seriously ill patients (due to an underlying pathology) with extremely low vitamin D levels could be predisposed to COVID-19 infection and increased COVID-19 severity and mortality. Furthermore, 25-OHD levels are the used biomarker of vitamin D status in the study population, nevertheless, they correlate poorly with the active form of vitamin D (1,25-OH2D), which exerts the effects of vitamin D on a cellular level. Thus, this study cannot exclude effects of 1,25-OH2D on COVID-19 risk.

Another limitation of this cohort relates to the fact that not all participants have been tested for present (or past) COVID-19 infection; consequentially, taking participants who were not tested as controls could be a potential source of bias, given that misclassification of controls might be substantial due to the presence of asymptomatic infected individuals, further driving our findings to the null. This is evident from the 1:2 ratio between outpatient vs. inpatient cases. It should be acknowledged that the COVID-19 cases in UK biobank have a high rate of hospitalisation due to the very limited and targeted testing at this stage of the pandemic in the UK, so this study reflects mainly those with more severe COVID-19 and gives less information about true infection risk, or risk of milder disease. In addition, we excluded individuals with a negative COVID-19 testing result from the controls due to the risk of those being false negatives. Although there is a risk of introducing selection bias, we believe that the risk of introducing misclassification bias if we included them in the analysis could be higher^22,23^. Additionally, given the presence of asymptomatic infected individuals, taking participants who were not tested as controls could also be another potential source of bias. Our study assessed the effect of genetically predicted vitamin D levels on COVID-19 risk while taking into consideration of ambient UVB radiation during the pandemic. We show an indication of an inverse association between genetically predicted vitamin D levels and severe COVID-19. Findings from our study are consistent with a recent randomised controlled trial (RCT) that found protective effect of vitamin D supplementation among those hospitalised with COVID-19^24^. However, other clinical trials did not show an effect. For instance, a randomised trial of 240 patients showed that supplementation with a single very large dose of 200,000 IU of vitamin D_3_ that increased serum vitamin D levels (21–44 ng/ml) was nonetheless ineffective in decreasing the length of hospital stay or any other clinical outcomes among hospitalized patients with severe COVID-19^25^. It has been estimated that one SD change in standardized natural-log transformed 25-OHD levels corresponds to a change in 25-OHD levels of 29.2 nmol/l in vitamin D insufficient individuals (serum 25-OHD levels < 50 nmol/l), which is comparable to the 21.2 nmol/l mean increase in 25-OHD levels conferred by taking daily 400 IU of cholecalciferol, the amount of vitamin D most often found in vitamin D supplements^26^. This estimation has clinical implication on the dose of vitamin D supplement for disease prevention. Given the lack of highly effective therapies against COVID-19, it is important to remain open-minded to emerging results from rigorously conducted studies of vitamin D.

In conclusion, we found no significant associations between COVID-19 risk and measured 25-OHD levels after adjusted for covariates, but this finding is limited by the fact that the vitamin D levels were measured on average 11 years before the pandemic. Ambient UVB was strongly and inversely associated with COVID-19 hospitalization and death. The main MR analysis did not show that genetically-predicted vitamin D levels were causally associated with COVID-19 risk, although MR sensitivity analyses indicated a potential causal effect. Overall, the effect of vitamin D levels on the risk or severity of COVID-19 remains controversial, further studies are needed to validate vitamin D supplementation as a means of protecting against worsened COVID-19.

## Supplementary Information

Supplementary Information.

## Data Availability

Data used in this study were obtained from UK Biobank under an approved data request application (application ID: 10775).

## Abbreviations

25-OHD: 25-Hydroxyvitamin D
BMI: Body mass index
COVID-19: Corona Virus Disease 2019
IQR: Interquartile range
MR: Mendelian randomisation
OR: Odds ratio
SD: Standard deviation
UVB: Ultraviolet radiation b
vitD: Vitamin D
wGRS: Weighted genetic risk score
