# Supplementary material for: An observational and Mendelian randomisation study on vitamin D and COVID-19 risk in UK Biobank
Source: Sci Rep. 2021 Sep 14;11:18262. doi: 10.1038/s41598-021-97679-5 (PMC8440633; doi:10.1038/s41598-021-97679-5)
Supplement: Supplementary file 1 — Supplementary Information. [file 41598_2021_97679_MOESM1_ESM.docx]

**Title:** An observational and Mendelian randomisation study on vitamin D and COVID-19 risk in UK Biobank

**Authors:** Xue Li^1,2^, Jos van Geffen^3^, Michiel van Weele^3^, Xiaomeng Zhang^2^, Yazhou He^2^, Xiangrui Meng^4^, Maria Timofeeva^5,6^, Harry Campbell^2^, Malcolm Dunlop^5^, Lina Zgaga^7§^, Evropi Theodoratou^2,8§^

**Affiliations**

^1^ School of Public Health and the Second Affiliated Hospital, Zhejiang University School of Medicine, Hangzhou, China

^2^ Centre for Global Health, Usher Institute, University of Edinburgh, Edinburgh, Scotland

^3^ Royal Netherlands Meteorological Institute (KNMI), De Bilt, the Netherlands.

^4^ Vanke School of public Health,Tsinghua University, Beijing 10084, China

^5^ Colon Cancer Genetics Group, Cancer Research UK Edinburgh Centre and Medical Research Council Human Genetics Unit, Medical Research Council Institute of Genetics and Molecular Medicine, University of Edinburgh, Edinburgh, United Kingdom

^6^ DIAS, Danish Institute for Advanced Study, Department of Public Health, University of Southern Denmark, Odense, Denmark

^7^ Department of Public Health and Primary Care, Institute of Population Health, Trinity College Dublin, Republic of Ireland.

^8^ Cancer Research UK Edinburgh Centre, MRC Institute of Genetics and Molecular Medicine, University of Edinburgh, Edinburgh, Scotland

^§^ Corresponding authors

Evropi Theodoratou, Centre for Global Health, Usher Institute, University of Edinburgh, Edinburgh, United Kingdom, [e.theodoratou@ed.ac.uk](mailto:e.theodoratou@ed.ac.uk), (+44) 0131 650 3210

Lina Zgaga, Department of Public Health and Primary Care, Institute of Population Health, Trinity College Dublin, Republic of Ireland, [ZGAGAL@tcd.ie](mailto:ZGAGAL@tcd.ie)

***Supplementary Methods***

*Vitamin D status attributable to dermal synthesis (vitD-UVB)*

To account for the vitamin D status attributable to dermal synthesis (vitD-UVB), we calculated the cumulative and weighted vitD-UVB dose from the TEMIS database, version 2.0 (http://www.temis.nl/uvradiation/UVdose.html), as it has been done previously (1-3). More details on the method can be found elsewhere (1-3). Briefly, we extracted daily UVB dose at wavelengths that induces vitamin D synthesis at each participant’s residential location over 135 days preceding the date of diagnosis for cases. Dates were randomly allocated to controls, from the distribution that was identical to that observed in cases. We weighted the daily UVB contributions before summing them up because more recent UVB exposure contributes more than exposures from a more distant past, since vitamin D is being synthesized and used up.

*Mendelian randomization (MR)*

Mendelian randomization (MR) design provides a cost-effective analogy to a randomized controlled trial by using genetic variants as proxies to test the causality of an association between exposure (vitamin D levels) and outcome (COVID-19) of interest. Here we applied inverse-variance weighted MR approach as the main analysis, and the simple mode, Egger, weighted median and weighted mode as sensitivity analyses to explore the robustness of the findings (4, 5). Details of these MR approaches, including their different assumptions, are explained as below.

Mean-based methods: The inverse variance weighted (IVW) MR and Egger MR provide two mean-based estimators. The IVW MR approach assumes that variants exhibit no horizontal pleiotropy, while Egger regression relaxes the horizontal pleiotropy assumption further by allowing a non-zero intercept which essentially allows overall horizontal pleiotropy to be directional, where its total effect influences the outcome in a specific direction. Egger regression further allows heterogeneity around the slope having accounted for overall directional horizontal pleiotropy, as long as the horizontal pleiotropy effects are not correlated with the SNP-exposure effects (also known as the INSIDE assumption).

Median-based methods: This analytical approach takes the median effect of all available instruments. The sample median method requires that half the instruments need to be valid to obtain unbiased estimate. The weighted median method allows stronger instruments to contribute more towards the estimate and obtain an estimate by weighting the contribution of each instrument by the inverse of its variance.

Mode-based methods: The mode-based estimator clusters the instruments into groups based on similarity of causal effects, and returns the final causal effect estimate based on the cluster that has the largest number of instruments. This provide two mode-based estimators: the simple mode is the unweighted mode of the empirical density function of causal estimates, the weighted mode is weighted by the inverse variance of the outcome effect.

MR-PRESSO: The MR-PRESSO is applied for the global test, outlier test, and distortion test using the MR pleiotropy residual sum and outlier (MR-PRESSO) R package. Specifically, the global test detects horizontal pleiotropy among the MR instruments; the outlier test corrects for horizontal pleiotropy via outlier removal; the distortion test identifies significant distortion in the causal estimates before and after outlier removal.

A summary of MR analytical approaches and their assumptions

| Method type | Methods | Assumptions |
| --- | --- | --- |
| Mean-based methods | IVW^*^ | No horizontal pleiotropy |
|  | Egger^*^ | Balanced horizontal pleiotropy after accounting for directional pleiotropy |
| Median-based methods | Weighted median | At least half the weight of the instruments is due to valid instruments. |
| Mode-based methods | Simple mode | After clustering instruments by causal estimates, the largest cluster is correct, weighting by the exposure variance. |
|  | Weighted mode | After clustering instruments by causal estimates, the largest cluster is correct, weighting by the exposure and outcome variances. |

^*^Abbreviations: IVW: inverse variance weighted

References

1. O'Sullivan F, Laird E, Kelly D, van Geffen J, van Weele M, McNulty H, et al. Ambient UVB Dose and Sun Enjoyment Are Important Predictors of Vitamin D Status in an Older Population. The Journal of nutrition. 2017;147(5):858-68.

2. O'Sullivan F, Raftery T, van Weele M, van Geffen J, McNamara D, O'Morain C, et al. Sunshine is an Important Determinant of Vitamin D Status Even Among High-dose Supplement Users: Secondary Analysis of a Randomized Controlled Trial in Crohn's Disease Patients. Photochemistry and photobiology. 2019;95(4):1060-7.

3. O'Sullivan F, van Geffen J, van Weele M, Zgaga L. Annual Ambient UVB at Wavelengths that Induce Vitamin D Synthesis is Associated with Reduced Esophageal and Gastric Cancer Risk: A Nested Case-Control Study. Photochemistry and photobiology. 2018;94(4):797-806.

4. Burgess S, Scott RA, Timpson NJ, Davey Smith G, Thompson SG. Using published data in Mendelian randomization: a blueprint for efficient identification of causal risk factors. European journal of epidemiology. 2015;30(7):543-52.

5. Hemani G, Zheng J, Elsworth B, Wade KH, Haberland V, Baird D, et al. The MR-Base platform supports systematic causal inference across the human phenome. eLife. 2018;7.

**Supplementary Tables**

Table S1 Associations between vitamin D and confirmed COVID-19 risk in White individuals*.

|  | **COVID positive (N=1524) *vs* controls (N=415,596)** | | **COVID hospitalization (N=883) *vs* non-hospitalization (N=497) cases** | | **COVID death (N=357) *vs* non-death (N=1023) cases** | |
| --- | --- | --- | --- | --- | --- | --- |
|  | OR (95% CI) | p-val | OR (95% CI) | p-val | OR (95% CI) | p-val |
| vitD (nmol/L) | 1.00 (0.99-1.01) | 0.273 | 1.00 (0.99-1.01) | 0.665 | 1.00 (0.99-1.01) | 0.796 |
| vitD_May_adjusted (nmol/L) | 1.00 (0.99-1.01) | 0.287 | 1.00 (0.99-1.01) | 0.532 | 1.00 (0.99-1.01) | 0.806 |
| vitD_categorical |  |  |  |  |  |  |
| 0-25 nmol/L | ref | -- | ref | -- | ref | -- |
| 25-50 nmol/L | 0.92 (0.77-1.10) | 0.355 | 0.65 (0.42-1.01) | 0.057 | 0.77 (0.50-1.18) | 0.229 |
| 50 nmol/L | 0.82 (0.68-0.99) | 0.036 | 0.62 (0.41-0.94) | 0.024 | 0.75 (0.48-1.16) | 0.191 |
| vitD-wGRS_134_^§^ | 0.89 (0.78-1.01) | 0.079 | 1.04 (0.79-1.36) | 0.782 | 1.31 (0.94-1.83) | 0.117 |
| vitD-UVB | 1.00 (0.99-1.01) | 0.984 | 0.98 (0.97-0.99) | <2×10^-16^ | 0.98 (0.97-0.99) | 4.93×10^-16^ |
| vitD-wGRS_134_ + vitD-UVB† |  |  |  |  |  |  |
| vitD-wGRS_134_ | 0.90 (0.79-1.02) | 0.108 | 0.86 (0.64-1.16) | 0.233 | 1.19 (0.84-1.70) | 0.332 |
| vitD-UVB | 1.00 (0.99-1.01) | 0.860 | 0.98 (0.97-0.99) | <2×10^-16^ | 0.98 (0.97-0.99) | 1.47×10^-14^ |

^*^Multivariable regression with adjustment for age, gender, body mass index (BMI), month of blood draw (adjusted for vitD and vitD-categorical only), physical activity, smoking and alcohol status, sunshine exposure variables (i.e., time spend outdoors in summer, time spent outdoors in winter and the use of sun/uv protection), vitamin D supplement intake, deprivation index, and comorbidities of CVDs, diabetes, asthma, and malignancy.

^§^Multivariable regression was additionally adjusted for the first 20 genetic principal components (PCs) and genotype panel.

†Multivariable regression was fitted by including both vitD-wGRS_134_ and vitD-UVB in the same model to examine the effects of genetically predicted vitamin D levels and ambient UVB jointly.

Table S2 Associations between Vitamin D and the infection, hospitalization and death of COVID-19 stratified by BMI (Normal [BMI<25kg/m^2^], Obese/overweight [≥25kg/m^2^]).*

|  | **COVID positive (N=1746) vs controls (N=415,596)** | | | | **COVID hospitalization (N=1020) vs non- hospitalization (N=576) cases** | | | | **COVID death (N=399) vs non-death (N=1347) cases** | | | |
| --- | --- | --- | --- | --- | --- | --- | --- | --- | --- | --- | --- | --- |
|  | Normal BMI | | Obese/overweight | | Normal | | Obese/overweight | | Normal | | Obese/overweight | |
|  | OR (95% CI) | p-val | OR (95% CI) | p-val | OR (95% CI) | p-val | OR (95% CI) | p-val | OR (95% CI) | p-val | OR (95% CI) | p-val |
| vitD (nmol/L) | 0.99 (0.98-1.00) | 0.063 | 1.00 (0.99-1.01) | 0.493 | 1.00 (0.99-1.01) | 0.505 | 1.00 (0.99-1.01) | 0.998 | 0.99 (0.98-1.01) | 0.568 | 1.00 (0.99-1.01) | 0.858 |
| vitD_May_adjusted (nmol/L) | 0.99 (0.98-1.00) | 0.067 | 1.00 (0.99-1.01) | 0.587 | 1.00 (0.99-1.01) | 0.630 | 1.00 (0.99-1.01) | 0.847 | 0.99 (0.98-1.01) | 0.406 | 1.00 (0.99-1.01) | 0.840 |
| vitD_categorical^†^ |  |  |  |  |  |  |  |  |  |  |  |  |
| 0-25 nmol/L | ref | -- | ref | -- | ref | -- | ref | -- | ref | -- | ref | -- |
| 25-50 nmol/L | 0.87 (0.64-1.19) | 0.379 | 0.98 (0.83-1.16) | 0.832 | 0.79 (0.39-1.60) | 0.506 | 0.90 (0.61-1.33) | 0.613 | 0.79 (0.31-2.04) | 0.633 | 0.72 (0.46-1.12) | 0.142 |
| 50 nmol/L | 0.78 (0.57-1.08) | 0.132 | 0.88 (0.73-1.06) | 0.171 | 1.05 (0.52-2.12) | 0.899 | 0.85 (0.56-1.29) | 0.442 | 0.80 (0.32-2.01) | 0.631 | 0.74 (0.46-1.16) | 0.192 |
| vitD-wGRS_134_^§^ | 0.94 (0.72-1.21) | 0.615 | 0.89 (0.77-1.04) | 0.142 | 1.77 (0.99-3.13) | 0.051 | 0.86 (0.64-1.18) | 0.395 | 1.02 (0.43-2.44) | 0.957 | 1.31 (0.92-1.88) | 0.135 |
| vitD-UVB | 1.00 (0.99-1.01) | 0.249 | 1.00 (0.99-1.01) | 0.102 | 0.98 (0.97-0.99) | 1.44×10^-9^ | 0.98 (0.97-0.99) | <2×10^-16^ | 0.98 (0.97-0.99) | 0.001 | 0.98 (0.97-0.99) | 4.01×10^-15^ |
| vitD-wGRS_134_ + vitD-UVB† |  |  |  |  |  |  |  |  |  |  |  |  |
| vitD-wGRS_134_ | 0.94 (0.73-1.22) | 0.642 | 0.90 (0.78-1.04) | 0.150 | 1.40 (0.76-2.58) | 0.273 | 0.73(0.52-1.03) | 0.071 | 0.78 (0.31-1.94) | 0.588 | 1.19 (0.82-1.72) | 0.306 |
| vitD-UVB | 1.00 (0.99-1.01) | 0.191 | 1.00 (0.99-1.01) | 0.150 | 0.98 (0.97-0.99) | 1.84×10^-8^ | 0.98 (0.97-0.99) | <2×10^-16^ | 0.98 (0.97-0.99) | 0.006 | 0.98 (0.97-0.99) | 2.05×10^-13^ |

*Multivariable regression with adjustment for age, gender, month of blood draw (adjusted for vitD and vitD-categorical only), ethnicity, physical activity, smoking and alcohol status, sunshine exposure variables (i.e., time spend outdoors in summer, time spent outdoors in winter and the use of sun/uv protection), vitamin D supplement intake, deprivation index, and comorbidities of CVDs, diabetes, asthma, and malignancy.

^§^Multivariable regression was additionally adjusted for the first 20 genetic principal components (PCs) and genotype panel.

†Multivariable regression was fitted by including both vitD-wGRS_134_ and vitD-UVB in the same model to examine the effects of genetically predicted vitamin D levels and ambient UVB jointly.

Table S3 Associations between Vitamin D and confirmed COVID-19 risk in Asian individuals.*

|  | **COVID positive (N=78) *vs* controls (N=9538)** | | **COVID hospitalization (N=56) *vs* non- hospitalization (N=22) cases** | | **COVID death (N=11) *vs* non-death (N=66) cases** | |
| --- | --- | --- | --- | --- | --- | --- |
|  | OR (95% CI) | *p-val* | OR (95% CI) | *p-val* | OR (95% CI) | *p-*val |
| vitD (nmol/L) | 0.99 (0.98-1.02) | 0.835 | 0.96 (0.86-1.07) | 0.467 | 1.03 (0.94-1.21) | 0.558 |
| vitD_May_adjusted (nmol/L) | 1.00 (0.99-1.02) | 0.425 | 0.99 (0.95-1.04) | 0.806 | 1.03 (0.94-1.12) | 0.532 |
| vitD_categorical |  |  |  |  |  |  |
| 0-25 nmol/L | ref | -- | ref | -- | ref | -- |
| 25-50 nmol/L | 1.00 (0.56-1.77) | 0.987 | 4.26 (0.61-29.85) | 0.145 | inf | -- |
| 50 nmol/L | 1.22 (0.49-3.10) | 0.116 | 0.27 (0.02-4.09) | 0.344 | inf | -- |
| vitD-wGRS_134_^§^ | 1.21 (0.62-2.40) | 0.557 | 0.49 (0.06-4.15) | 0.511 | 0.20 (0.01-138.22) | 0.630 |
| vitD-UVB | 1.00 (0.99-1.01) | 0.415 | 0.98 (0.97-0.99) | 0.044 | 0.86 (0.61-1.21) | 0.395 |
| vitD-wGRS_134_ + vitD-UVB† |  |  |  |  |  |  |
| vitD-wGRS_134_ | 1.34 (0.68-2.67) | 0.395 | 0.41 (0.05-3.48) | 0.412 | 0.05 (0.01-12.36) | 0.301 |
| vitD-UVB | 1.00 (0.99-1.01) | 0.584 | 0.98 (0.97-1.00) | 0.052 | 0.94 (0.81-1.05) | 0.281 |

^*^Multivariable regression with adjustment for age, gender, BMI, month of blood draw (adjusted for vitD and vitD-categorical only), physical activity, smoking and alcohol status, sunshine exposure variables (i.e., time spend outdoors in summer, time spent outdoors in winter and the use of sun/uv protection), vitamin D supplement intake, deprivation index, and comorbidities of CVDs, diabetes, asthma, and malignancy.

^§^Multivariable regression was additionally adjusted for the first 20 genetic principal components (PCs) and genotype panel.

†Multivariable regression was fitted by including both vitD-wGRS_134_ and vitD-UVB in the same model to examine the effects of genetically predicted vitamin D levels and ambient UVB jointly.

Table S4 Associations between Vitamin D and confirmed COVID-19 risk in Black individuals.*

|  | **COVID positive (N=45) *vs* controls (N=3854)** | | **COVID hospitalization (N=28) *vs* non- hospitalization (N=17) cases** | | **COVID death (N=12) *vs* non-death (N=33) cases** | |
| --- | --- | --- | --- | --- | --- | --- |
|  | OR (95% CI) | *p-val* | OR (95% CI) | *p-val* | OR (95% CI) | *p-*val |
| vitD (nmol/L) | 0.99 (0.97-1.02) | 0.634 | 1.04 (0.96-1.12) | 0.369 | 0.99 (0.92-1.06) | 0.760 |
| vitD_May_adjusted (nmol/L) | 1.00 (0.98-1.02) | 0.731 | 1.08 (0.98-1.18) | 0.116 | 1.00 (0.92-1.09) | 0.972 |
| vitD_categorical |  |  |  |  |  |  |
| 0-25 nmol/L | ref | -- | ref | -- | ref | -- |
| 25-50 nmol/L | 0.62 (0.31-1.24) | 0.175 | 2.78 (0.30-25.74) | 0.367 | 0.41 (0.04-4.72) | 0.476 |
| 50 nmol/L | 0.88 (0.38-2.26) | 0.789 | 0.52 (0.01-25.59) | 0.743 | 2.61 (0.10-75.64) | 0.576 |
| vitD-wGRS_134_^§^ | 1.48 (0.37-5.76) | 0.568 | 1.14 (0.01-209.04) | 0.958 | inf | 0.251 |
| vitD-UVB | 0.98 (0.97-0.99) | 0.001 | 1.00 (0.98-1.02) | 0.884 | 0.98 (0.94-1.02) | 0.318 |
| vitD-wGRS_134_ + vitD-UVB† |  |  |  |  |  |  |
| vitD-wGRS_134_ | 1.67 (0.42-6.67) | 0.466 | 3.92 (0.01-1469) | 0.593 | inf | 0.309 |
| vitD-UVB | 0.98 (0.97-0.99) | 0.001 | 0.98 (0.94-1.04) | 0.223 | 0.98 (0.92-1.06) | 0.771 |

^*^Multivariable regression with adjustment for age, gender, BMI, month of blood draw (adjusted for vitD and vitD-categorical only), physical activity, smoking and alcohol status, sunshine exposure variables (i.e., time spend outdoors in summer, time spent outdoors in winter and the use of sun/uv protection), vitamin D supplement intake, deprivation index, and comorbidities of CVDs, diabetes, asthma, and malignancy.

^§^Multivariable regression was additionally adjusted for the first 20 genetic principal components (PCs) and genotype panel.

†Multivariable regression was fitted by including both vitD-wGRS_134_ and vitD-UVB in the same model to examine the effects of genetically predicted vitamin D levels and ambient UVB jointly.

Table S5 Interactions between vitD-wGRS_134_, vitD-UVB and VDR in response to COVID-19 risk (White ancestry).*

| **Interactions** | **COVID positive (N=1524) *vs* controls (N=415,596)** | | **COVID hospitalization (N=883) *vs* non-hospitalization (N=497) cases** | | **COVID death (N=357) *vs* non-death (N=1023) cases** | |
| --- | --- | --- | --- | --- | --- | --- |
|  | beta | p-val | beta | p-val | beta | p-val |
| **vitD-wGRS_134_ and VDR** |  |  |  |  |  |  |
| vitD-wGRS_134_ | 0.158 | 0.382 | -0.225 | 0.560 | 0.675 | 0.146 |
| vitD-wGRS_134_: rs2228570_G | -0.103 | 0.289 | 0.122 | 0.559 | -0.425 | 0.114 |
| vitD-wGRS_134_: rs1544410_T | 0.328 | 0.488 | 0.357 | 0.747 | -1.921 | 0.124 |
| vitD-wGRS_134_: rs11568820_T | 0.006 | 0.959 | 0.122 | 0.600 | 0.096 | 0.735 |
| vitD-wGRS_134_: rs7975232_A | -0.264 | 0.067 | -0.610 | 0.058 | 0.210 | 0.583 |
| vitD-wGRS_134_: rs731236_G | -0.153 | 0.743 | 0.575 | 0.589 | 0.180 | 0.125 |
| **vitD-UVB and VDR** |  |  |  |  |  |  |
| vitD-UVB | -0.017 | 0.005 | -0.026 | 5.03e-10 | -0.013 | 0.017 |
| vitD-UVB: rs2228570_G | -0.004 | 0.633 | 0.002 | 0.404 | -0.001 | 0.519 |
| vitD-UVB: rs1544410_T | -0.011 | 0.303 | -0.008 | 0.424 | -0.010 | 0.466 |
| vitD-UVB: rs11568820_T | 0.003 | 0.389 | -0.003 | 0.289 | 0.002 | 0.477 |
| vitD-UVB: rs7975232_A | 0.001 | 0.824 | 0.004 | 0.282 | 0.001 | 0.909 |
| vitD-UVB: rs731236_G | 0.009 | 0.529 | 0.010 | 0.329 | 0.006 | 0.606 |

^*^Multivariable regression with adjustment for age, gender, BMI, physical activity, smoking and alcohol status, sunshine exposure variables (i.e., time spend outdoors in summer, time spent outdoors in winter and the use of sun/uv protection), vitamin D supplement intake, deprivation index, and comorbidities of CVDs, diabetes, asthma, and malignancy, the first 20 genetic principal components (PCs) and genotype panel.

Table S6 Interactions between vitD-wGRS_134_ and vitD-UVB in response to COVID-19 risk (White ancestry).

|  | **Model without Interaction *** | | | | **Model with Interaction*** | | | | | |
| --- | --- | --- | --- | --- | --- | --- | --- | --- | --- | --- |
| COVID-19 | vitD-wGRS_134_ | | vitD-UVB | | vitD-wGRS_134_ | | vitD-UVB | | interaction | |
|  | beta | p-val | beta | p-val | beta | p-val | beta | p-val | beta | p-val |
| Positive vs controls | -0.085 | 0.191 | -0.0001 | 0.511 | 0.249 | 0.055 | -0.001 | 0.155 | -0.005 | 0.001 |
| Hospitalization vs non-hospitalization | -0.125 | 0.391 | -0.020 | < 2×10^-16^ | -0.055 | 0.896 | -0.020 | < 2×10^-16^ | -0.001 | 0.642 |
| death vs non-death | 0.116 | 0.508 | -0.016 | 4.35×10^-16^ | -0.037 | 0.916 | -0.015 | 8.03×10^-11^ | 0.003 | 0.521 |

^*^Multivariable regression with adjustment for age, gender, BMI, physical activity, smoking and alcohol status, sunshine exposure variables (i.e., time spend outdoors in summer, time spent outdoors in winter and the use of sun/uv protection), vitamin D supplement intake, deprivation index, and comorbidities of CVDs, diabetes, asthma, and malignancy, the first 20 genetic principal components (PCs) and genotype panel.

Table S7 Association between vitD-wGRS_134_ and COVID-19 risk, stratified by vitD-UVB (White ancestry).*

|  | **COVID positive (N=1524) *vs* controls (N=415,596)** | | **COVID hospitalization (N=883) *vs* non-hospitalization (N=497) cases** | | **COVID death (N=357) *vs* non-death (N=1023) cases** | |
| --- | --- | --- | --- | --- | --- | --- |
| vitD-UVB | vitD-wGRS_134_ | | vitD-wGRS_134_ | | vitD-wGRS_134_ | |
|  | beta | p-val | beta | p-val | beta | p-val |
| Tertile 1 | 0.186 | 0.141 | -0.159 | 0.718 | 0.219 | 0.451 |
| Tertile 2 | -0.173 | 0.125 | -0.101 | 0.695 | 0.080 | 0.796 |
| Tertile 3 | -0.321 | 0.003 | -0.223 | 0.349 | 0.356 | 0.459 |

^*^Multivariable regression with adjustment for age, gender, BMI, physical activity, smoking and alcohol status, sunshine exposure variables (i.e., time spend outdoors in summer, time spent outdoors in winter and the use of sun/uv protection), vitamin D supplement intake, deprivation index, and comorbidities of CVDs, diabetes, asthma, and malignancy, the first 20 genetic principal components (PCs) and genotype panel.

Table S8 MR analyses of 134 vitD SNPs on COVID-19 infection (White ancestry).^†^

| **MR methods** | **Beta (se)** | **OR (95%CI)** | **p-val** |
| --- | --- | --- | --- |
| MR IVW | -0.25 (0.18) | 0.77 (0.55-1.11) | 0.160 |
| MR Egger* | -0.31 (0.22) | 0.73 (0.48-1.13) | 0.165 |
| Weighted median | -0.48 (0.20) | 0.61 (0.42-0.92) | 0.016 |
| Simple mode | -0.09 (0.43) | 0.91 (0.39-2.12) | 0.835 |
| Weighted mode | -0.33 (0.14) | 0.72 (0.55-0.95) | 0.021 |
| MR-PRESSO | -0.22 (0.10) | 0.80 (0.66-0.98) | 0.030 |

**^†^** The genetic associations between vitamin D SNPs and COVID-19 infection risk were estimated with adjustment for age, sex, the first 20 genetic PCs and genotype panel.

* There is no observed horizontal pleiotropy for the MR Egger analysis (p__pleiotropy_ = 0.644).
